# Supplementary material for: Optimization of CRISPR/Cas9 Gene Editing System in Sheep (Ovis aries) Oocytes via Microinjection
Source: Int J Mol Sci. 2025 Jan 26;26(3):1065. doi: 10.3390/ijms26031065 (PMC11817664; doi:10.3390/ijms26031065)
Supplement: Supplementary file 1 [file ijms-26-01065-s001.zip › ijms-3385300-supplementary.pdf]

Supplemental figures

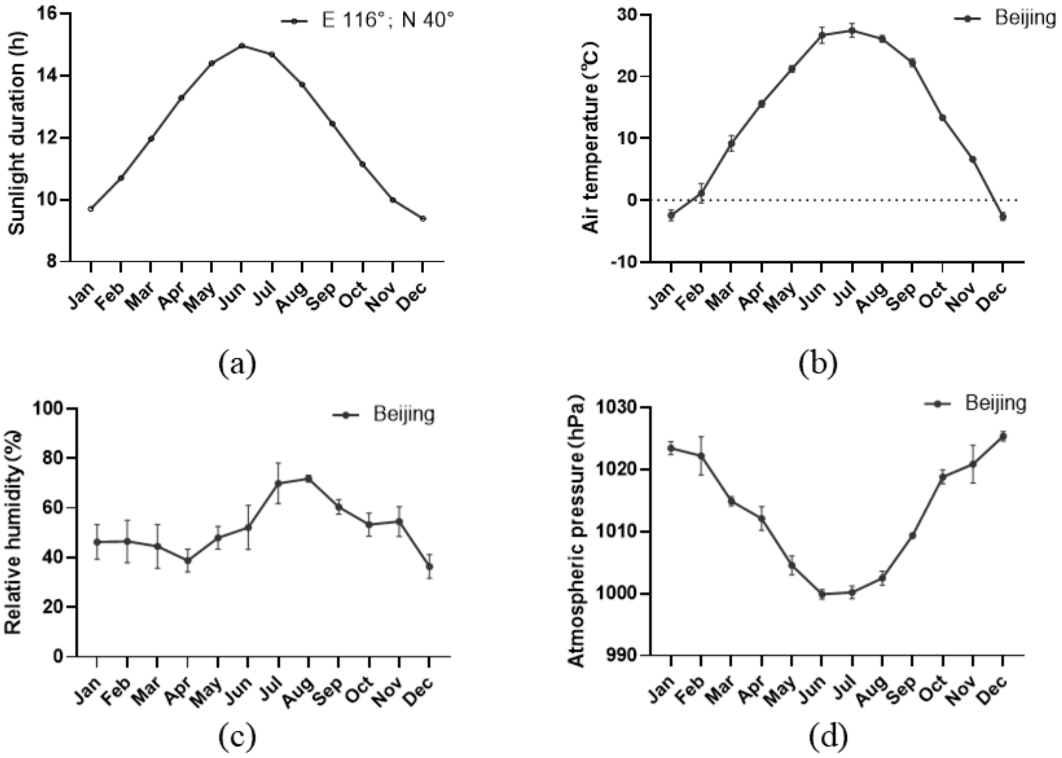

Figure S1. Seasonal variations in climate of Beijing. (a) Mean sunlight duration across months in Beijing; (b) Mean air temperature across months in Beijing; (c) Mean relative humidity across months in Beijing; (d) Mean atmospheric pressure across months in Beijing.

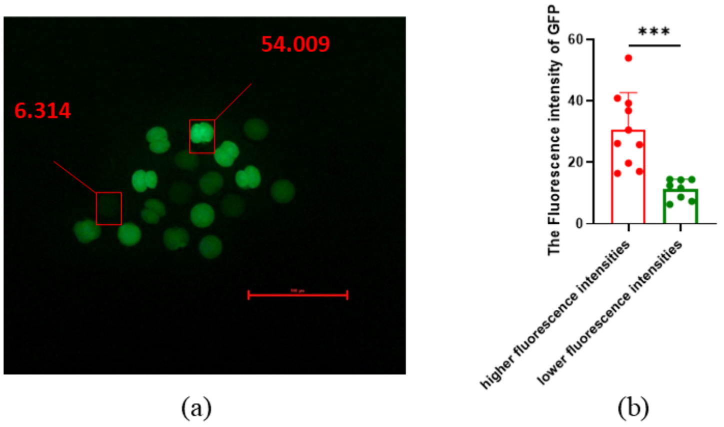

Figure S2. Editing efficiency in oocytes post-microinjection based on fluorescent intensity. (a) Variation in GFP fluorescent intensity of oocytes post-microinjection; (b) Significant differences between low and high fluorescent intensity oocytes. All data are presented as mean ± standard deviation (SD). For oocytes with higher fluorescence intensity, *n*=10; for those with lower fluorescence intensity, *n*=8. The difference is highly significant (*** P < 0.001).

(a)

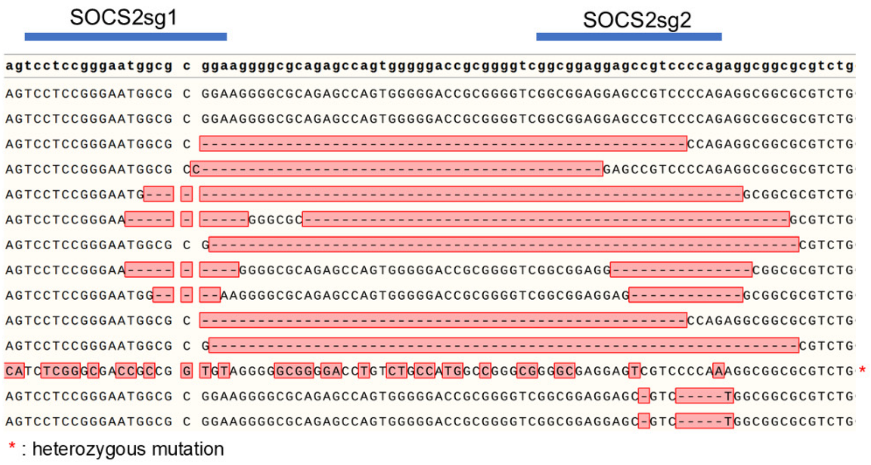
SOCS2sg1
SOCS2sg2
* : heterozygous mutation

(b)

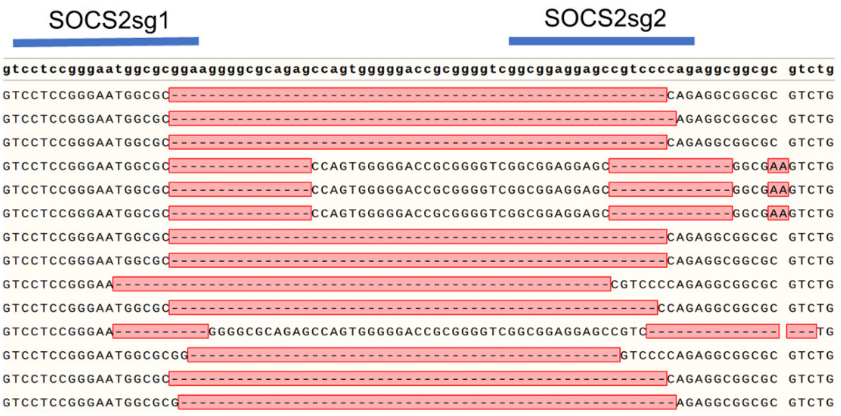
SOCS2sg1
SOCS2sg2

(c)

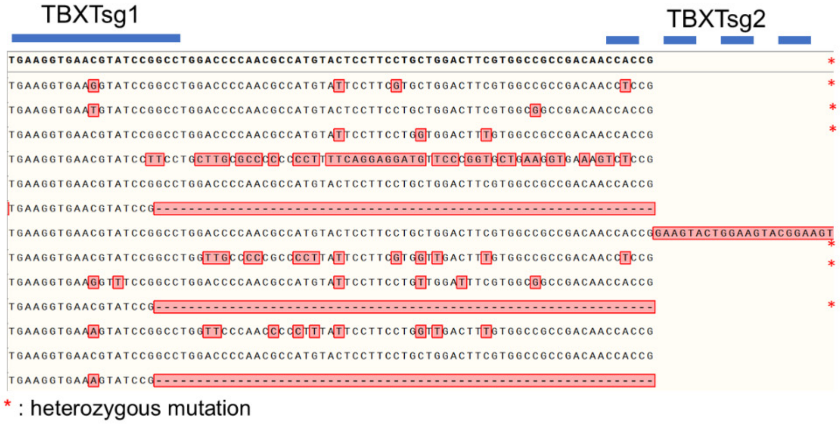
TBXTsg1
TBXTsg2
* : heterozygous mutation

(d)

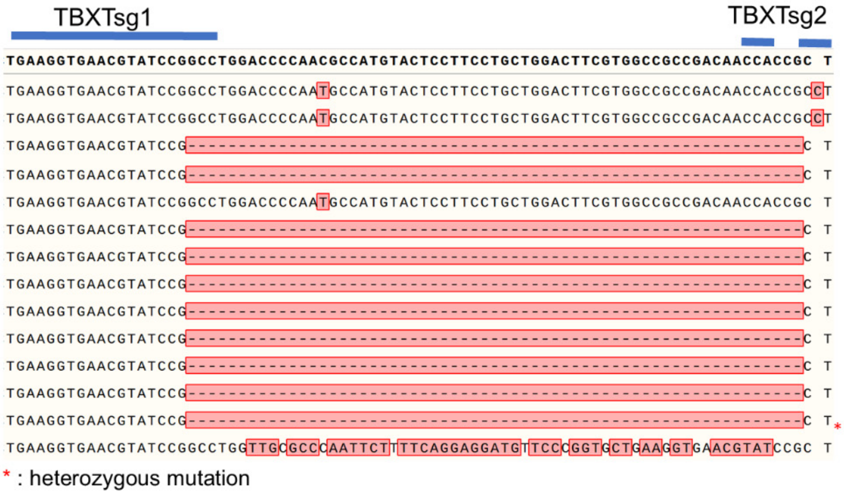
TBXTsg1
TBXTsg2
* : heterozygous mutation

Figure S3. Genotype analysis of edited oocytes using Cas9 protein or mRNA. (a) Genotype of edited oocytes using Cas9 mRNA and *SOCS2* sgRNA; (b) Genotype of edited oocytes using Cas9 protein and *SOCS2* sgRNA; (c) Genotype of edited oocytes using Cas9 mRNA and *TBXT* sgRNA; (d) Genotype of edited oocytes using Cas9 protein and *TBXT* sgRNA.

Supplemental Tables

Table S1. Spearman's rank correlation coefficient of climate parameter in seasons

Climate parameter	Season	r_s(95%CI)	*P*
air temperature	spring	0.149(-0.256, 0.51)	0.457
	summer	-0.659(-0.906, -0.078)	0.027
	autumn	0.032(-0.454, 0.503)	0.900
	winter	-0.056(-0.622, 0.549)	0.863
sunlight duration	spring	0.167(-0.239, 0.523)	0.406
	summer	-0.659(-0.906, -0.078)	0.027
	autumn	0.079(-0.416, 0.537)	0.757
	winter	0.190(-0.447, 0.699)	0.554
atmospheric pressure	spring	-0.013(-0.401, 0.379)	0.950
	summer	0.009(-0.607, 0.619)	0.978
	autumn	-0.032(-0.503, 0.454)	0.900
	winter	-0.212(-0.711, 0.428)	0.508
relative humidity	spring	0.005(-0.386, 0.394)	0.980
	summer	0.697(0.147, 0.918)	0.017
	autumn	0.193(-0.314, 0.615)	0.442
	winter	-0.056(-0.622, 0.549)	0.863

Table S2. Editing efficiency of different target gene by using of Cas9 protein or mRNA

Target gene	*SOCS2* editing efficiency		*TBXT* editing efficiency	
Cas9 protein	homozygous	Total	homozygous	Total
	79.41%±13.75%	90.63%±10.97%	43.41%±30.46%	76%±16.27%
Cas9 mRNA	homozygous	Total	homozygous	Total
	48.89%±1.92%	74.28%±11.16%	3.33%±5.77%	62%±20.26%

Table S3. sg sequences targeted to genes in this study

Target gene	Exon	Sequence (5′-3′)
SOCS2sg1	E1	TCCTCCGGGAATGGCGGCGGA
SOCS2sg2	E1	GGCGGAGGAGCCGTCCCCAG
DYAsg1	E1	GAAGAAAGCTCTGATTCTGA
DYAsg2	E1	TGTGGAGGTGAAGACATCGT
TBXTsg1	E2	GAAGGTGAACGTATCCGGC
TBXTsg2	E2	CGTTCACGTACTTCCAGCGG

Table S4. primers of targeting genes in this study

Target gene	Name	Sequence (5′-3′)	Production

			size
SOCS2	SOCS2F1	CTCTCTGTCCTTTCTCTGGAACG	759bp
	SOCS2R1	GATTTCCAAGGAACCCTCCTCG	
	SOCS2NGSF	CTAAGAAGGACGTGTGCCCTTC	217bp
	SOCS2NGSR	CTACCTGTGTGACTGAGTTCCCT	
DYA	DYAF1	AGCTTTACTCTTTGATGATTCCTC	960bp
	DYAR1	CATGCTATACTTGGGCATTACG	
	DYANGSF	CTCTTTAGACCACCTTCCTGGT	248bp
	DYANGSR	CCACCTGAGCCACCATACTCCAATATA	
TBXT	TBXTF1	TGCGCCCCTTCCTTTTCAG	520bp
	TBXTR1	AGGCTAGGACGCAGAAGTTC	

Table S5. Genotypes of lambs analyzed by NGS sequencing.

Gene	Sheep	Genotype code	Edited genotype	reads number	Ratio
DYA	0471	0471-1	52D	4127	54.68%
		1471-2	3D，47D	3421	45.33%
	0472	0472-1	15D，17D	3990	53.90%
		0472-2	15D，3D	3412	46.09%
	0473	0473-1	52D	4383	58.44%
		0473-2	24D，1D	3117	41.56%
	0474	0474-1	10D	2026	89.53%
		0474-2	57D	237	10.47%
	0475	0475-1	53D	4954	65.27%
		0475-2	5D，7D	2636	34.73%
SOCS2	0463	0463-1	52D	3043	63.25%
		0463-2	2D,12D	1768	36.75%
	0464	0464-1	64D	2912	58.83%
		0464-2	10D	2038	41.17%
	0465	0465-1	65D	3207	69.76%
		0465-2	10D，30D	746	16.23%
		0465-3	10D，1D，3D	644	14.01%
	0466	0466-1	10D	6155	85.34%
		0466-2	10D	1057	14.66%
	0467	0467-1	1I	2796	52.11%
		0467-2	53D	2410	44.92%
		0467-3	1I，12D	159	2.96%
	0468	0468-1	65D	3452	87.00%
		0468-2	61D	516	13.00%

Table S6. Commercial recombinant CRISPR/Cas proteins.

Name	Catalog number	Company	Prduction Details
TrueCut Cas9 protein v2	A36496/7/8/9	ThermoFisher	recombinant Streptococcus pyogenes Cas9 (S.p Cas9) protein
TrueCut™ HiFi Cas9 Protein	A50574/5/6//7	ThermoFisher	engineered high fidelity Cas9 protein
CTS™ TrueCut™	A45220/1	ThermoFisher	recombinant S.p Cas9 protein

Cas9 Prote			
Guide-it Recombinant Cas9	632640/1; 632678/9	TaKaRa	recombinant S.p Cas9 protein
EnGen® Spy Cas9 HF1	M0667M/T-	NEW ENGLAND Biolabs (NEB)	Mutant of S.p Cas9 protein (N497A/R661A/Q695A/Q926A)
EnGen® Spy Cas9 NLS	M0646T/M	NEB	S.p Cas9 protein with NLS (nuclear localization signal) and 6×His tag at N-terminal
EnGen® Sau Cas9	M0654T	NEB	recombinant Staphylococcus aureus Cas9 with 6×His tag at C-terminal
EnGen® Seq1 Cas9	M0668T	NEB	recombinant Streptococcus equinus Cas9 with N- and C-terminal Simian virus 40 (SV40) T antigen NLS and a C-terminal 6×His tag
EnGen® Lba Cas12a(Cpf1)	M0653S/T	NEB	recombinant Lachnospiraceae bacterium ND2006 Cas12a
Spy Cas9	M0386S/T/M	NEB	recombinant S.p Cas9 protein
EnGen® Spy Cas9 nickase	M0650S/M	NEB	Mutant of recombinant S.p Cas9 protein(D10A)
EnGen® Spy dCas9	M0652S/T	NEB	Recombinant S.p. dCas9
Alt-R S.p.Cas9 Nuclease V3	108158/9; 10000735	Integrated DNA Technologies (IDT)	recombinant S.p Cas9 protein with NLS and C-terminal 6-His tag
Alt-R S.p.Cas9 V3, glycerol-free	10007806/7/8	IDT	recombinant S.p. Cas9 protein, glycerol-free with NLS and C-terminal 6-His tag
Alt-R S.p. HiFi Cas9 Nuclease V3	108160/1;10007803	IDT	high fidelity S.p. Cas9 protein with NLS and C-terminal 6-His tag
Alt-R S.p.Cas9-GFP V3	10008100/1	IDT	recombinant S.p Cas9 protein with GFP with NLS and C-terminal 6-His tag
Alt-R S.p.Cas9-RFP V3	10008162/3	IDT	recombinant S.p Cas9 protein with RFP with NLS and C-terminal 6-His tag
Alt-R S.p.Cas9 D10A nickase V3	1081062/3	IDT	Recombinant S.p. Cas9 nickase(D10A) with NLS and C-terminal 6-His tag
Alt-R S.p.Cas9 DH840A nickase V3	1081064/5	IDT	Recombinant S.p. Cas9 nickase(H840A) with NLS and C-terminal 6-His tag
Alt-R dCas9	1081066/7	IDT	Recombinant S.p. dCas9 with NLS and

protein V3			C-terminal 6-His tag
Alt-R A.s. Cas12a(cpf1) V3	1081068/9	IDT	Recombinant Acidaminococcus sp. BV3L6 (A.s.) nuclease with NLS and C-terminal 6-His tag
Alt-R A.s. Cas12a(cpf1)Ultra	10001272/3; 10007804	IDT	Recombinant A.s. nuclease with NLS and C-terminal 6-His tag
Alt-R L.b. Cas12a(cpf1)Ultra	10007922/3/4	IDT	Recombinant Lachnospiraceae bacterium ND2006 (L.b.) nuclease with NLS and C-terminal 6-His tag
Cas9 Nuclease	D0511	Beyotime	recombinant S.p Cas9 protein
